# The precision of two alternative indirect workflows for digital model production: an illusion or a possibility?

**DOI:** 10.1007/s00784-023-04996-2

**Published:** 2023-04-13

**Authors:** Noha Mohamed Elkersh, Rania A. Fahmy, Mohamed K. Zayet, Yousria S. Gaweesh, Mohamed G. Hassan

**Affiliations:** 1 Oral Medicine, Periodontology, Oral Diagnosis and Oral Radiology Department, Faculty of Dentistry, Alexandria University, Alexandria, Egypt; 2 Oral and Maxillofacial Radiology Department, Cairo University, Newgiza University, Cairo, Egypt; 3 Department of Orthodontics, Faculty of Dentistry, Assiut University, Assiut, Egypt; 4 Division of Bone and Mineral Diseases, Department of Medicine, School of Medicine, Washington University in St. Louis, St. Louis, MO USA

**Keywords:** Precision, Digital, CBCT, Desktop scanning, Digital model, Intraoral scanner

## Abstract

**Objective:**

Despite the clear drive from both research and clinical dentistry toward digital transformation, there are limitations to implementing intra-oral scanning (IOS) into daily dental practice. This study aimed to compare the precision of digital models obtained from two alternative indirect workflows to direct IOS.

**Material and methods:**

Two indirect digital workflows were evaluated in this study. In the IOS group (direct), IOS directly obtained digital impressions of participants’ upper and lower dental arches, while in the Scan Impression (Scan Imp) group (indirect), a desktop scanner scanned silicone-based impressions of upper and lower whole arches that were taken with plastic trays. In the cone-beam computed tomography impression (CBCT Imp) group (indirect), a CBCT machine scanned the silicone-based impressions. Then, the precision of the entire arch and individual teeth for all digital impressions was virtually quantified. Following superimposition, differences between standard tessellation language (STL) files obtained from both—direct and indirect—methods were evaluated by color-mapping and measuring the surface distance between superimposed STL files. Furthermore, 18 linear measurements were taken from each digital model. ANOVA with repeated measures, Pearson coefficient, and intraclass correlation coefficient were used for intergroup comparisons.

**Results:**

The digital models obtained from the two indirect workflows differed from the IOS in some dental and intra-arch measurements but were considered clinically acceptable. Ranked against IOS, CBCT Imp models had greater precision, followed by Scan Imp.

**Conclusion:**

Digital models obtained from two indirect, alternative workflows, desktop, and CBCT scanning of impression, have clinically acceptable accuracy and reliability of tooth size and intra-arch measurements, providing the use of proper methodologies.

**Clinical relevance:**

There are some limitations to implementing IOS in daily clinical practice. However, several alternative digital model production techniques might provide an affordable solution. Although they may insignificantly differ in accuracy, all can be applied clinically.

**Supplementary Information:**

The online version contains supplementary material available at 10.1007/s00784-023-04996-2.

## Introduction

In the last decade, biomedical innovations and digital technologies have been crucial in dental research and personalized dentistry. Currently, digital images, models, and radiography are widely used for oral and dental diagnosis, treatment planning, and management in daily clinical practices [1]. Digital models are considered an accurate, time-efficient alternative to the plaster models made from conventional impressions, directly affecting the quality of patient care [2]. Although considered the gold standard in many dental procedures, the impression-making process for plaster models is often uncomfortable for many patients. It presents challenges, especially when dealing with infants and/or patients with cleft palates. Moreover, previous studies showed that plaster models and impression materials wear with repeated measurements [3] are altered when exposed to different humidity and temperature levels and require additional storage space since patient dental records must be kept for a minimum of 7 to 10 years [4].

Recent advancements have made it possible to construct dental prostheses using a computerized process [5]. The IOS is a crucial component in the digital impression-making workflow. IOSs use an intraoral camera to capture dental arch features. The chairside use of computer-aided design and computer-aided manufacturing (CAD/CAM) is a typical example of digital workflow. This process does not require physical or impression castings and can produce restoration at a single clinical visit [6]. Moreover, many studies have proven the high accuracy and reliability of digital models obtained directly from IOSs, even in full arch scans [7, 8].

Currently, three commonly used workflows produce digital dental models for patients. The first is the direct workflow in which digital impressions are taken via an intraoral scanner (IOS). The second is an indirect workflow in which impressions are taken by scanning a silicone-based dental cast via a desktop scanner. This automatic 3D acquisition device quickly converts three-dimensional objects into 3D digital files using a beam of light or laser as a non-contact data-capturing tool [9, 10]. Finally, the third is an indirect workflow that uses CBCT patients’ scans to extract the required data to generate digital models [11]. CBCT scans of patient impressions and casts are an alternative to intraoral or desktop scanners without exposing the patient to an extra step.

Although there is a clear drive from the clinical dental community toward digital modeling through IOSs, there are limitations to implementing IOS in daily dental practice, including the price of IOSs, especially in low-income countries [12, 13]. Understanding these limitations and providing affordable, reliable alternatives will boost digital technologies’ adoption in daily dental practice. Therefore, this study aimed to compare the accuracy of full-arch digital dental models generated indirectly from desktop scanners and CBCT scanning of silicone-based impressions against those produced directly from IOSs. Both indirect methods showed clinically acceptable accuracy and reproducibility levels compared to the IOS. Furthermore, considering the level of precision, CBCT scanning of silicone-based impressions showed a general trend of greater precision in both single-tooth and intra-arch measurements.

## Material and methods

### Study settings and sample population

**Fig. 1:**
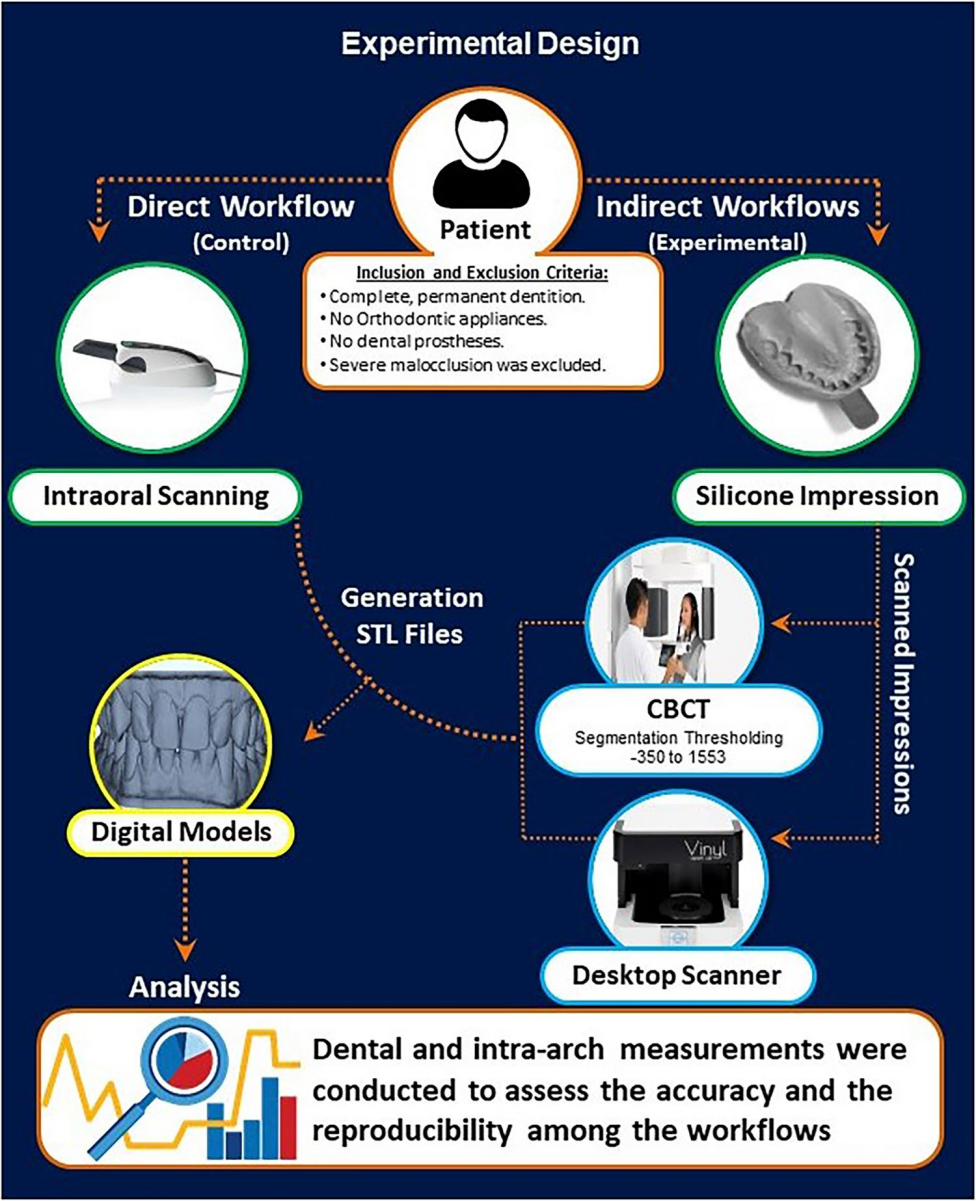
Study workflow. The process of producing three-dimensional digital models via the three direct and indirect workflows included in the current study

This study was designed and conducted according to the institutional review board guidelines of the Faculty of Dentistry, Alexandria University. The approval was obtained from the research ethics committee at the Faculty of Dentistry, Alexandria University (Ethics Committee no: IORG0008839). All participants were asked to sign an informed consent ahead of data collection. The sample size was estimated based on a 95% confidence level and 80% study power and was calculated using G*power (version 3.1.9.7). Eighteen participants—from the Alexandria University Dental School outpatient clinic—were randomly selected and enrolled in this study. The inclusion criteria were complete, permanent dentition without orthodontic appliances or dental prostheses. Participants with moderate to severe malocclusion and/or craniofacial anomalies were excluded from this study (Fig. 1).

### Generation of digital models

Digital models obtained from three workflows were measured and compared (Fig. 1). The first group (control), the IOS workflow, represented the control group. Bimaxillary intraoral scanning was performed for all participants using CS 3600 (Carestream Dental, Atlanta, GA, USA) and CS Scan Flow (v1.0.3) acquisition software. The same scanning protocol was used in all the participants as indicated by the manufacturer. Briefly, the occlusal surface of the dentition was kept dry to avoid any interference with the scanning process. The scanning started from the occlusal and incisal surfaces of teeth as a reference and starting points, followed by buccal and labial surfaces, and finally, lingual and palatal surfaces. STL monochromatic files were obtained directly from the software, although colored PLY files can be obtained from the intraoral scanner. This process was applied for all files to standardize the file format for all study groups (*n* = 36 STL files). This group is referred to as the IOS group (Fig. 1).

For the second group, the desktop scanner workflow, silicone-based two-step impressions (Silaxil, Lascod, Italy), were used to record the participant’s upper and lower dentitions. First, the impressions were sprayed with an anti-glare spray (IP Scan-Spray, IP Divison, Germany). Next, the borders were carefully trimmed to remove any overhangs that may interfere during the scanning process. Then, the impressions were scanned with a desktop scanner (Smart optics Vinyl Open Air, Sensortechnik, GmbH, Italy) using a digital impression scan module. This scanner applies the principle of structured light active triangulation with white light LED, using a 1.3 Megapixel camera of 6 μm accuracy, with a measurement field of 80 × 60 × 85 mm. The scanned impressions were exported from the laboratory CAD-CAM software as STL files (*n* = 36 STL files). This group is referred to as Scan Imp [12] (Fig. 1).

For the last group, the CBCT workflow, the same silicone-based two-step impressions were scanned using a CBCT machine (Veraview × 800, JMorita, Japan) (100 kV, 8 mA, 0.08 × 0.08 × 0.08 mm voxel size, and 80 × 40 mm field of view) within 6 h. Next, Digital Imaging and Communication in Medicine (DICOM) files were imported into a 3D imaging program Mimics software (Materialise NV, Leuven, Belgium, version 19.0) to create a new mask. Finally, segmentation and thresholding using a consistent grayscale (− 350–1553) were performed to convert DICOM files into STL files (*n* = 36 STL files). This group is referred to as CBCT Imp [13] (Fig. 1).

### Data collection

**Fig. 2:**
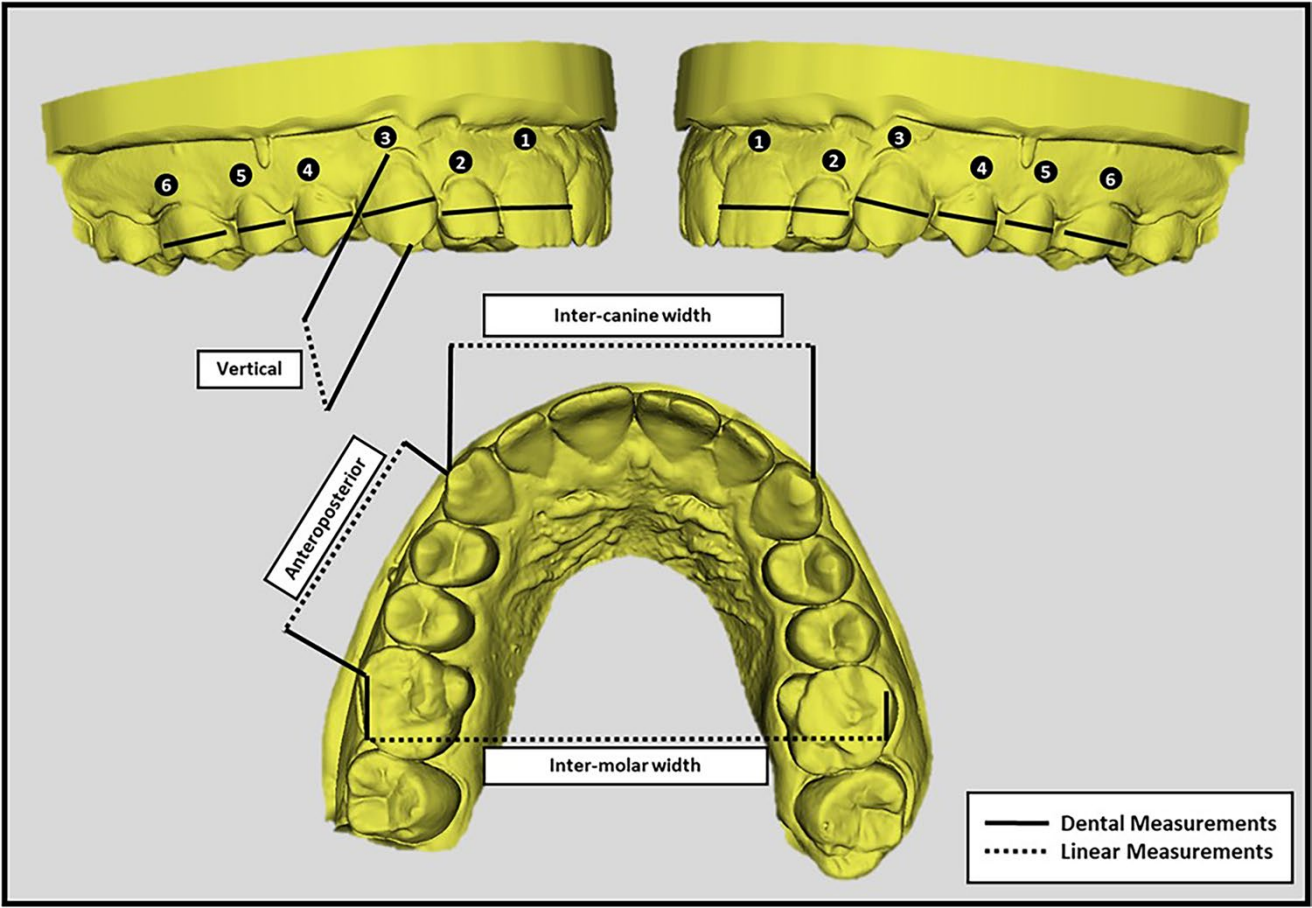
Measurements made on the digital cast models (maxillary arch)

A total of 108 maxillary and mandibular digital models from the three different workflows (IOS, Scan Imp, and CBCT Imp) were measured with 1728 linear measurements. The measurements of the digital models were all obtained using 3D-Slicer software (version 5.0.2). Following the STL file import, each model was aligned parallel in all directions. For all teeth (twelve measurements for each arch), first molar to first molar, in both arches, the tooth measurements included the mesiodistal widths from point contact to point contact (crown width) (Fig. 2). Moreover, four linear measurements were made on each digital model in the three dimensions: anteroposterior (the canine cusp tip to the mesiobuccal cusp tip of the first molar on the right side), transverse (inter-canine and inter-molar), and vertical (the canine height, which was measured from the cusp tip to the gingival margin of the right canine by a line parallel to the long axis of the tooth) (Fig. 2).

A 3D deviation analysis was done between the digital models of each participant and the reference using 3-Matics software (Materialise NV, Belgium, Version 11.0). Each two STL models were imported into the software, “*N* points registration” tool was used to align the two models together roughly by selecting eight corresponding points; then, the “Global registration” tool was used and set to one hundred iterations to improve the registration process with the least possible error. The borders of the two models were trimmed together about 5 mm from the gingival margin to reduce errors. The mean deviation difference (measured in mm) was calculated by the “Analyse” tool present in the software.

Color maps were visualized to identify the spots of local deviation. They were set for a range of ± 0.5 mm (3 color segments). The white zone indicates a good fit, the red zone (0.25–0.5 mm) indicates positive errors, and the blue zone (− 0.5– − 0.25 mm) indicates negative errors.

### Statistical analysis

First, descriptive statistics for teeth linear measurements and intra-arch distances have been described using mean and standard deviation. Second, the collected data were tested for normality by the Shapiro–Wilk test. To assess the accuracy, ANOVA with repeated measures was used to compare the digital models obtained from the three workflows. Subsequently, a post hoc analysis (Bonferroni adjusted) for pairwise comparison between every two workflows has been conducted to verify for which workflow the teeth and intra-arch distances were statistically significant. The Pearson coefficient was used to correlate between every two workflows, while intra class correlation coefficient was used for agreement.

Two observers (NME and MGH) independently located the teeth linear measurements and the intra-arch distances on seven randomly selected STL files to determine inter-observer reproducibility. The ICC was used to determine the inter-observer reliability of the repeated measurement errors (Supplemental Tables 1 and 5). For all statistical analyses, the significance level was set at *p* < 0.05. Statistical data analysis was performed using the IBM SPSS software package (version 20.0).

## Results

Mean dental, intra-arch linear measurements, and their standard deviations are listed in Tables 1 and 4. The mean differences between every two workflows indicating the mean imprecision are listed in Supplemental Tables 2 and 3. The ICC and Pearson coefficients highlighting the reproducibility are listed in Tables 2, 3, 5, and 6.

**Table 1:** Comparison between the three workflows (IOS, Scan Imp, CBCT Imp) in relation to dental measurements

	IOS (mm) (*n* = 18)	Scan Imp (mm) (*n* = 18)	CBCT Imp (mm) (*n* = 18)	*F*	*p*	Sig. bet. devices		
						p_1_	p_2_	p_3_
Maxillary								
R6	9.57^a^ ± 0.73	9.41^a^ ± 0.85	9.49^a^ ± 0.72	2.975	0.064	NS	NS	NS
R5	5.78^ab^ ± 0.73	5.77^a^ ± 0.65	5.57^b^ ± 0.55	4.896	0.014^*^	NS	NS	0.041^*^
R4	5.83^a^ ± 0.47	5.66^a^ ± 0.53	5.75^a^ ± 0.49	2.885	0.070	NS	NS	NS
R3	6.69^a^ ± 0.94	6.66^a^ ± 0.86	6.51^a^ ± 0.84	.952	0.375	NS	NS	NS
R2	6.16^a^ ± 0.50	6.03^a^ ± 0.67	6.01^a^ ± 0.52	2.465	0.100	NS	NS	NS
R1	8.10^a^ ± 0.87	7.89^b^ ± 0.86	7.90^ab^ ± 0.97	3.789	0.033^*^	0.011^*^	NS	NS
L1	8.04^a^ ± 0.89	7.93^a^ ± 0.85	7.92^a^ ± 1.0	3.430	0.054	NS	NS	NS
L2	6.11^a^ ± 0.46	5.89^b^ ± 0.53	5.99^ab^ ± 0.54	5.658	0.008^*^	0.031^*^	NS	NS
L3	6.84^a^ ± 0.92	6.72^ab^ ± 0.99	6.40^b^ ± 0.92	3.924	0.047^*^	NS	0.034^*^	NS
L4	5.86^a^ ± 0.49	5.72^a^ ± 0.50	5.76^a^ ± 0.39	2.820	0.092	NS	NS	NS
L5	5.85^a^ ± 0.72	5.77^a^ ± 0.65	5.68^a^ ± 0.58	2.524	0.095	NS	NS	NS
L6	9.49^a^ ± 0.85	9.22^b^ ± 0.78	9.30^ab^ ± 0.61	5.412	0.009^*^	0.001^*^	NS	NS
Mandibular								
R6	9.50^a^ ± 0.71	9.37^a^ ± 0.71	9.44^a^ ± 0.71	1.569	0.229	NS	NS	NS
R5	6.61^a^ ± 1.18	6.57^a^ ± 1.32	6.52^a^ ± 1.33	.661	0.523	NS	NS	NS
R4	5.96^a^ ± 0.38	5.79^a^ ± 0.44	5.94^a^ ± 0.40	2.884	0.070	NS	NS	NS
R3	5.89^a^ ± 0.51	5.77^a^ ± 0.52	5.80^a^ ± 0.52	1.205	0.296	NS	NS	NS
R2	5.50^a^ ± 0.34	5.30^a^ ± 0.46	5.42^a^ ± 0.30	3.402	0.059	NS	NS	NS
R1	5.10^a^ ± 0.37	4.89^b^ ± 0.32	5.0^ab^ ± 0.30	4.044	0.027^*^	0.017^*^	NS	NS
L1	5.08^a^ ± 0.39	4.83^b^ ± 0.37	4.88^b^ ± 0.35	10.268^*^	0.002^*^	< 0.001^*^	0.003^*^	NS
L2	5.45^a^ ± 0.40	5.22^b^ ± 0.53	5.40^a^ ± 0.39	4.817	0.014^*^	0.018^*^	NS	0.046^*^
L3	5.71^a^ ± 0.51	5.57^a^ ± 0.56	5.69^a^ ± 0.51	1.895	0.166	NS	NS	NS
L4	5.86^a^ ± 0.49	5.79^a^ ± 0.61	5.81^a^ ± 0.51	0.528	0.595	NS	NS	NS
L5	6.54^a^ ± 1.38	6.44^a^ ± 1.37	6.42^a^ ± 1.37	3.004	0.063	NS	NS	NS
L6	9.40^a^ ± 0.75	9.38^a^ ± 0.78	9.37^a^ ± 0.77	0.088	0.916	NS	NS	NS

Data was expressed using mean ± SD. *SD* standard deviation, *NS* non-significant difference, *F F* test (ANOVA) with repeated measures, Sig. bet. periods was done using post hoc test (adjusted Bonferroni)

*p p* value for comparing between the studied devices

*p*_*1*_* p* value for comparing between IOS and Scan Imp

*p*_*2*_* p* value for comparing between IOS and CBCT Imp

*p*_*3*_* p* value for comparing between Scan imp and CBCT imp

^*^Statistically significant at *p* ≤ 0.05

**Table 2:** Correlation between different workflows in relation to the dental measurements (*n* = 18)

	IOS vs. Scan		IOS vs. CBCT		Scan vs. CBCT	
	*r*	*p*	*r*	*p*	*r*	*p*
Maxillary						
R6	0.955	< 0.001^*^	0.921	< 0.001^*^	0.949	< 0.001^*^
R5	0.928	< 0.001^*^	0.873	< 0.001^*^	0.878	< 0.001^*^
R4	0.825	< 0.001^*^	0.867	< 0.001^*^	0.766	< 0.001^*^
R3	0.908	< 0.001^*^	0.712	0.001^*^	0.655	0.003^*^
R2	0.907	< 0.001^*^	0.899	< 0.001^*^	0.851	< 0.001^*^
R1	0.952	< 0.001^*^	0.917	< 0.001^*^	0.902	< 0.001^*^
L1	0.976	< 0.001^*^	0.984	< 0.001^*^	0.970	< 0.001^*^
L2	0.798	< 0.001^*^	0.875	< 0.001^*^	0.902	< 0.001^*^
L3	0.916	< 0.001^*^	0.616	0.007^*^	0.671	0.002^*^
L4	0.943	< 0.001^*^	0.794	< 0.001^*^	0.797	< 0.001^*^
L5	0.939	< 0.001^*^	0.872	< 0.001^*^	0.853	< 0.001^*^
L6	0.951	< 0.001^*^	0.907	< 0.001^*^	0.832	< 0.001^*^
Mandibular						
R6	0.864	< 0.001^*^	0.971	< 0.001^*^	0.876	< 0.001^*^
R5	0.982	< 0.001^*^	0.966	< 0.001^*^	0.972	< 0.001^*^
R4	0.710	0.001^*^	0.760	< 0.001^*^	0.561	0.015^*^
R3	0.957	< 0.001^*^	0.697	0.001^*^	0.692	0.001^*^
R2	0.630	0.005^*^	0.768	< 0.001^*^	0.620	0.006^*^
R1	0.499	0.035^*^	0.626	0.005^*^	0.462	0.054
L1	0.865	< 0.001^*^	0.841	< 0.001^*^	0.607	0.008^*^
L2	0.726	0.001^*^	0.800	< 0.001^*^	0.750	< 0.001^*^
L3	0.824	< 0.001^*^	0.797	< 0.001^*^	0.836	< 0.001^*^
L4	0.863	< 0.001^*^	0.818	< 0.001^*^	0.937	< 0.001^*^
L5	0.986	< 0.001^*^	0.988	< 0.001^*^	0.987	< 0.001^*^
L6	0.948	< 0.001^*^	0.931	< 0.001^*^	0.916	< 0.001^*^

*r* Pearson coefficient

^*^Statistically significant at *p* ≤ 0.05

**Table 3:** Intra-class correlation coefficient for different workflows in relation to dental measurements (*n* = 18)

	IOS vs. scan			IOS vs. CBCT			Scan vs. CBCT		
	ICC	95% C.I (LL–UL)	p	ICC	95% C.I (LL–UL)	p	ICC	95% C.I (LL–UL)	*p*
Maxillary									
R6	0.928	0.769 – 0.975	< 0.001^*^	0.919	0.802 – 0.969	< 0.001^*^	0.936	0.840 – 0.975	< 0.001^*^
R5	0.926	0.812 – 0.972	< 0.001^*^	0.805	0.504 – 0.926	< 0.001^*^	0.826	0.500 – 0.937	< 0.001^*^
R4	0.781	0.456 – 0.916	< 0.001^*^	0.859	0.666 – 0.945	< 0.001^*^	0.763	0.480 – 0.904	< 0.001^*^
R3	0.909	0.773 – 0.965	< 0.001^*^	0.704	0.377 – 0.877	< 0.001^*^	0.656	0.294 – 0.855	0.001^*^
R2	0.857	0.655 – 0.944	< 0.001^*^	0.868	0.600 – 0.953	< 0.001^*^	0.832	0.605 – 0.934	< 0.001^*^
R1	0.927	0.673 – 0.977	< 0.001^*^	0.895	0.717 – 0.961	< 0.001^*^	0.901	0.754 – 0.962	< 0.001^*^
L1	0.968	0.892 – 0.989	< 0.001^*^	0.970	0.894 – 0.990	< 0.001^*^	0.960	0.896 – 0.985	< 0.001^*^
L2	0.726	0.307 – 0.897	< 0.001^*^	0.845	0.618 – 0.940	< 0.001^*^	0.892	0.728 – 0.958	< 0.001^*^
L3	0.911	0.781 – 0.965	< 0.001^*^	0.564	0.151 – 0.810	0.003^*^	0.645	0.281 – 0.849	0.001^*^
L4	0.908	0.564 – 0.972	< 0.001^*^	0.764	0.482 – 0.904	< 0.001^*^	0.779	0.502 – 0.911	< 0.001^*^
L5	0.931	0.829 – 0.974	< 0.001^*^	0.829	0.584 – 0.934	< 0.001^*^	0.845	0.641 – 0.939	< 0.001^*^
L6	0.897	0.367 – 0.971	< 0.001^*^	0.835	0.585 – 0.937	< 0.001^*^	0.811	0.568 – 0.924	< 0.001^*^
Mandibular									
R6	0.856	0.660 – 0.943	< 0.001^*^	0.969	0.920 – 0.988	< 0.001^*^	0.877	0.707 – 0.952	< 0.001^*^
R5	0.977	0.940 – 0.991	< 0.001^*^	0.959	0.896 – 0.984	< 0.001^*^	0.973	0.930 – 0.990	< 0.001^*^
R4	0.656	0.270 – 0.857	< 0.001^*^	0.769	0.478 – 0.907	< 0.001^*^	0.536	0.131 – 0.793	0.007^*^
R3	0.933	0.700 – 0.979	< 0.001^*^	0.698	0.364 – 0.875	< 0.001^*^	0.703	0.358 – 0.878	0.001^*^
R2	0.546	0.131 – 0.801	0.003^*^	0.750	0.457 – 0.898	< 0.001^*^	0.551	0.148 – 0.802	0.006^*^
R1	0.423	-0.002 – 0.730	0.016^*^	0.592	0.207 – 0.823	0.003^*^	0.448	0.016 – 0.746	0.024^*^
L1	0.712	-0.029 – 0.915	< 0.001^*^	0.735	0.142 – 0.912	< 0.001^*^	0.613	0.219 – 0.835	0.003^*^
L2	0.633	0.210 – 0.850	< 0.001^*^	0.803	0.553 – 0.921	< 0.001^*^	0.678	0.311 – 0.867	< 0.001^*^
L3	0.804	0.547 – 0.922	< 0.001^*^	0.806	0.550 – 0.923	< 0.001^*^	0.820	0.583 – 0.928	< 0.001^*^
L4	0.842	0.632 – 0.937	< 0.001^*^	0.821	0.586 – 0.929	< 0.001^*^	0.926	0.813 – 0.971	< 0.001^*^
L5	0.984	0.955 – 0.994	< 0.001^*^	0.985	0.949 – 0.995	< 0.001^*^	0.987	0.813 – 0.971	< 0.001^*^
L6	0.949	0.870 – 0.981	< 0.001^*^	0.934	0.833 – 0.975	< 0.001^*^	0.920	0.800 – 0.969	< 0.001^*^

*ICC* intraclass correlation coefficient, *CI* confidence interval, *LL* lower limit, UL upper limit

^*^Statistically significant at *p* ≤ 0.05

Dental measurements on the digital models taken from the three workflows are shown in Table 1. The Scan Imp and CBCT Imp groups differed from the IOS group by a maximum of 0.279 and 0.438 mm, respectively. A statistically significant difference (*p* ≤ 0.05) in linear measurements between the three workflows indicated a trend of greater imprecision in the maxillary dental arch. Some tooth size values (16 dental measurements) showed significant differences between the three workflows as maxillary R5 (*p* = 0.014), maxillary R1 (*p* = 0.033), maxillary L3 (*p* = 0.047), mandibular R1 (*p* = 0.027), and mandibular L1 (*p* = 0.02), and L2 (*p* = 0.014). The post hoc analysis revealed more significant differences in the dental measurements between the (IOS) and (Scan Imp) groups in comparison to the other pairwise comparisons (Table 1), highlighting a trend of higher imprecision.

Intra-arch measurement accuracy for all digital models is shown in Table 4. The maxillary anteroposterior, intercanine, and mandibular anteroposterior, intercanine, and Intermolar measurements were not significantly different between digital models obtained from the three workflows. However, like the dental measurements, there was a trend of greater imprecision in the maxillary dental arch.

**Table 4:** Comparison between the three workflows (IOS, Scan Imp, CBCT Imp) in relation to intra-arch measurements

	IOS (mm) (*n* = 18)	Scan imp (mm) (*n* = 18)	CBCT imp (mm) (*n* = 18)	*F*	*p*	Sig. bet. devices		
						p_1_	p_2_	p_3_
Maxillary								
Right AP dimension	23.22^a^ ± 4.03	23.0^a^ ± 4.04	23.24^a^ ± 3.95	2.217	0.142	NS	NS	NS
Inter-canine	34.97^a^ ± 1.98	34.70^a^ ± 1.78	34.93^a^ ± 1.75	2.342	0.133	NS	NS	NS
Inter-molar	52.35^a^ ± 3.28	51.97^b^ ± 3.07	52.27^a^ ± 3.04	7.710^*^	0.002^*^	0.004^*^	NS	0.020^*^
Right vertical dimension	9.08^a^ ± 1.15	8.69^b^ ± 1.13	8.50^b^ ± 1.03	8.928^*^	0.003^*^	0.042^*^	0.011^*^	NS
Mandibular								
Right AP dimension	25.92^a^ ± 4.59	25.64^a^ ± 4.70	25.71^a^ ± 4.71	2.309	0.115	NS	NS	NS
Inter-canine	26.57^a^ ± 2.63	26.09^a^ ± 2.36	26.33^a^ ± 2.35	3.710	0.053	NS	NS	NS
Inter-molar	47.09^a^ ± 4.93	46.67^a^ ± 4.87	46.98^a^ ± 4.78	1.824	0.193	NS	NS	NS
Right vertical dimension	8.96^a^ ± 1.51	8.71^ab^ ± 1.27	8.40^b^ ± 1.28	9.115^*^	0.001^*^	NS	0.006^*^	NS

Data was expressed using mean ± SD. *SD* standard deviation, *NS* non-significant difference, *F F* test (ANOVA) with repeated measures, Sig. bet. periods was done using post hoc test (adjusted Bonferroni)

*p p* value for comparing between the studied devices

*p*_*1*_* p* value for comparing between IOS and Scan imp

*p*_*2*_* p* value for comparing between IOS and CBCT imp

*p*_*3*_* p* value for comparing between Scan imp and CBCT imp

^*^Statistically significant at *p* ≤ 0.05

The ICC and Pearson coefficients were high for dental and intra-arch measurements, indicating that all measurements on digital models from all workflows were highly reproducible (Tables 2,3, 5, and 6). Tables 3 and 6 show the repeatability of dental and intra-arch measurements of STL files related to the IOS group. The reproducibility using IOS compared to Scan Imp and CBCT Imp highlighted a trend of higher precision.

**Table 5:** Correlation between different workflows in relation to intra-arch measurements (*n* = 18)

	IOS vs. Scan		IOS vs. CBCT		Scan vs. CBCT	
	*r*	*p*	*r*	*p*	*r*	*p*
Maxillary						
Right AP dimension	0.987	< 0.001^*^	0.990	< 0.001^*^	0.997	< 0.001^*^
Inter-canine	0.931	< 0.001^*^	0.959	< 0.001^*^	0.979	< 0.001^*^
Inter-molar	0.993	< 0.001^*^	0.992	< 0.001^*^	0.991	< 0.001^*^
Right vertical dimension	0.861	< 0.001^*^	0.786	< 0.001^*^	0.936	< 0.001^*^
Mandibular						
Right AP dimension	0.992	< 0.001^*^	0.991	< 0.001^*^	0.995	< 0.001^*^
Inter-canine	0.938	< 0.001^*^	0.954	< 0.001^*^	0.980	< 0.001^*^
Inter-molar	0.976	< 0.001^*^	0.969	< 0.001^*^	0.996	< 0.001^*^
Right vertical dimension	0.956	< 0.001^*^	0.903	< 0.001^*^	0.916	< 0.001^*^

*r* Pearson coefficient

^*^Statistically significant at *p* ≤ 0.05

**Table 6:** Intra-class correlation coefficient for different workflows in relation to intra-arch measurements each (*n* = 18)

	IOS vs. Scan			IOS vs. CBCT			Scan vs. CBCT		
	ICC	95% C.I (LL – UL)	*p*	ICC	95% C.I (LL – UL)	*p*	ICC	95% C.I (LL – UL)	*p*
Maxillary									
Right AP dimension	0.986	0.964 – 0.995	<0.001^*^	0.990	0.974 – 0.996	<0.001^*^	0.995	0.976 – 0.998	<0.001^*^
Inter-canine	0.920	0.798 – 0.969	<0.001^*^	0.955	0.884 – 0.983	<0.001^*^	0.972	0.898 – 0.991	<0.001^*^
Inter-molar	0.985	0.889 – 0.996	<0.001^*^	0.989	0.972 – 0.996	<0.001^*^	0.986	0.936 – 0.996	<0.001^*^
Right vertical dimension	0.820	0.489 – 0.935	<0.001^*^	0.693	0.187 – 0.887	<0.001^*^	0.921	0.787 – 0.971	<0.001^*^
Mandibular									
Right AP dimension	0.991	0.973 – 0.997	<0.001^*^	0.990	0.974 – 0.996	<0.001^*^	0.996	0.988 – 0.998	<0.001^*^
Inter-canine	0.919	0.769 – 0.970	<0.001^*^	0.947	0.866 – 0.980	<0.001^*^	0.975	0.925 – 0.991	<0.001^*^
Inter-molar	0.973	0.929 – 0.990	<0.001^*^	0.970	0.923 – 0.989	<0.001^*^	0.994	0.973 – 0.998	<0.001^*^
Right vertical dimension	0.931	0.803 – 0.975	<0.001^*^	0.831	0.364 – 0.946	<0.001^*^	0.894	0.681 – 0.962	<0.001^*^

*ICC*, intraclass correlation coefficient; *CI*, confidence interval; *LL*, lower limit; *UL*, upper limit

*Statistically significant at *p* ≤ 0.05

**Fig. 3:**
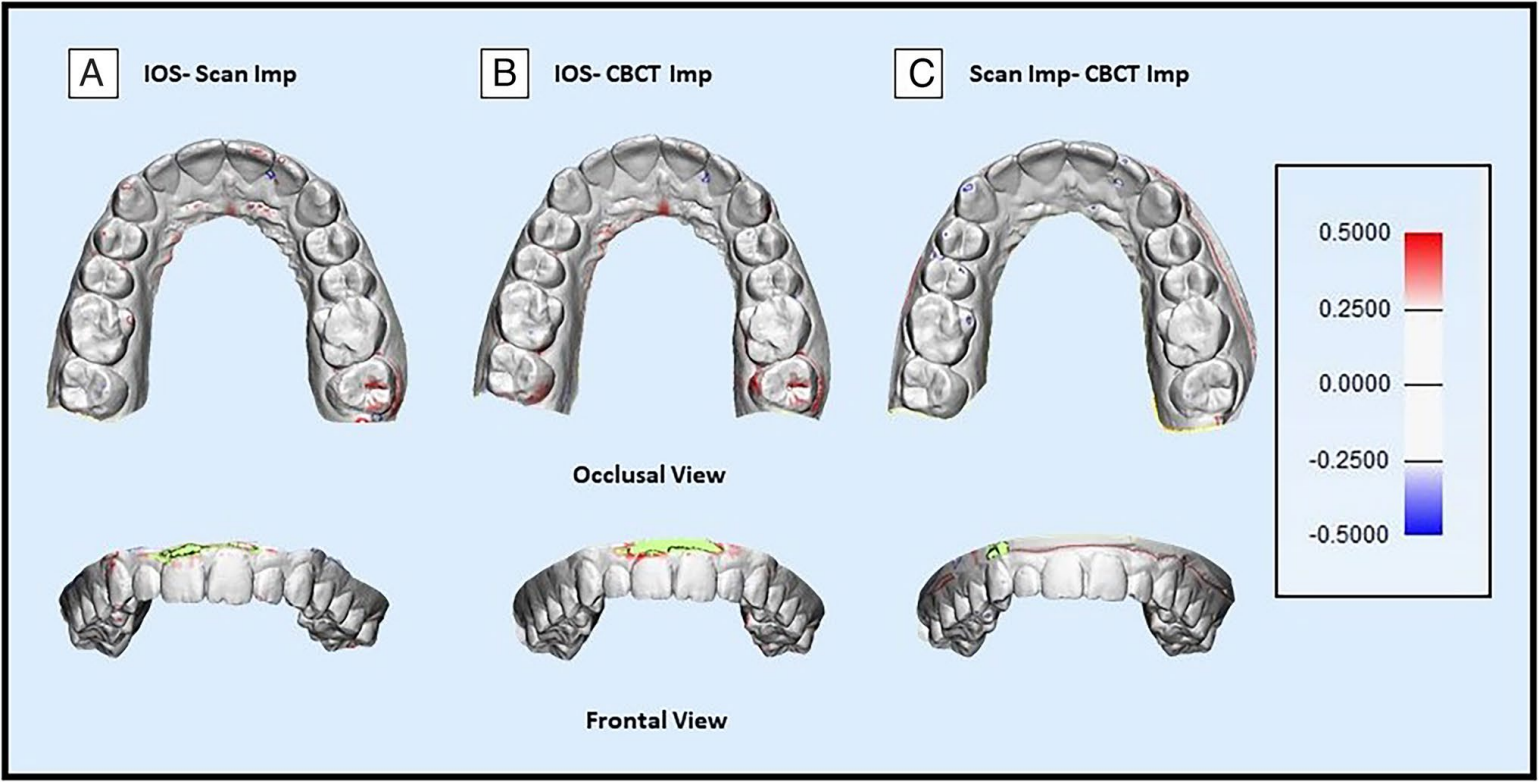
Occlusal and frontal views of the 3D comparison of the studied workflows. **A** 3D comparison of IOS with Scan Imp. **B** 3D comparison of IOS with CBCT Imp. **C** 3D comparison of Scan Imp with CBCT Imp. Red zone (0.25–0.5 mm), blue zone (− 0.5– − 0.25 mm), and green zone (out-of-bounds)

The mean deviation (mm) was calculated for all groups (Supplemental Table 4). The same trend resulting from the dental and intra-arch linear measurements is highlighted in the 3D heatmap visualization of the differences between the three workflows (Fig. 3); digital models obtained from Scan Imp showed a higher deviation than those obtained by the IOS. The regions of greatest discrepancy to the IOS were the molars on both sides (Fig. 3).

## Discussion

Despite the rapid implementation of digital technologies in everyday dental practice, little information is available about the limitations of using these tools, especially among specialists in developing countries; also, little data is available about the usefulness of other alternative techniques [14, 15]. This study investigates the precision of digital models obtained from two workflows that do not directly require a digital impression from the oral environment. We investigated the precision and the trueness of the measurements on digital models obtained from intra-oral scanning (IOS), impression scanning with a desktop scanner (Scan Imp), and CBCT impression scanning (CBCT Imp). To our knowledge, no studies have evaluated the digital models obtained from these workflows and how they could be optimized for use in the clinical environment.

We considered the digital models obtained from the intra-oral scanning as the control group; the intra-oral scanner used in this trial has been found to show high accuracy and reproducibility [8, 16, 17]. This consideration could be a valid point of debate [18, 19]. Many previous studies evaluated the accuracy of intraoral scanners in full arch scans [16, 17, 20–23]. Some studies showed that optical impressions have higher accuracy than conventional impressions and/or stone models [17, 20, 24]. For example, Wiranto et al*.* and Naidu and Freer reported an increase in dental measurements collected from digital impressions compared to stone models [25, 26]. In contrast, others have shown that conventional impression materials with high accuracy prove higher precision in full arch scans than all IOS systems. However, irreversible hydrocolloid or alginate impressions demonstrated lower precision than digital impressions [21–23].

Dental literature discussed extensively the trueness, precision, and accuracy of IOSs obtained from different devices and/or techniques [2, 24, 25, 27]. Trueness is the agreement between the arithmetical mean of multiple test results and the true or accepted reference, while precision refers to the agreement among test results [28]. Therefore, researchers used the superimposed digitized models to evaluate the accuracy, in which deviations between the two datasets can be visualized and measured three-dimensionally [28]. However, it is important to highlight that this method relies on the software ICP algorithm to fit the models on each other, which averages out the differences between them. For this reason, in this study, we collected 16 linear measurements besides the 3D deviation analysis to overcome this possible bias.

The results of this study show a trend toward clinically-acceptable accuracy between digital impressions from indirect CBCT and desktop scanner workflows and direct IOS workflows. However, dental and intra-arch measurements from digital models obtained from CBCT impression scanning showed higher accuracy. In contrast, those obtained from the digital impression scanning showed the lowest accuracy, as revealed by post hoc pairwise comparisons. Three-dimensional deviation analysis and heat mapping yielded similar findings to linear measurements. The CBCT Imp group showed less mean deviation than the IOS group, while the Scan imp showed a slightly higher deviation. Two systematic reviews explained that the linear measurements done on different digital models could vary, with mean differences between 0.04 and 0.4 mm [18, 19]. This was argued to be a clinically acceptable range. Many researchers compared measurements in stone and digital models and concluded that a comparable range of differences is clinically acceptable [18, 27, 29]. In the current study, the maximum deviation was 0.1 mm in the Scan Imp group, which is still considered clinically accepted.

Previous studies evaluated the accuracy of digital models produced from CBCT scanning of impressions [11, 30]. Wesemann et al. showed that the CBCT digitalization of impressions led to insufficient or inaccurate results [11]. In this study, we found better accuracy in both measurements (dental and intra-arch). This disagreement has many explanations; one regards the CBCT machine’s settings. Previous studies generated digital models using CBCT machines with higher voxel size than the one used in our study (0.08 mm) [11, 29–31]. Park et al. found no significant differences in most linear measurements between the digitization of impressions with CBCT and the scanning of stone casts with a desktop scanner. These results agree with our current study concluding that the digitalization of impressions with CBCT is adequate for use in clinical practice [30].

Many studies have explained the accuracy enhancement of CBCT scan models vs. laser-based scans [11, 13, 30–32]. This is because CBCT scans are volume scans, while desktop or laser-based scanners are surface scans. This explains why CBCT scans are less affected by the angle of irradiation or the object’s shape, which makes the CBCT scan advantageous in cases of crowding [30]. On the other hand, digital models generated from CBCT scanning have a granular surface texture, not as smooth as those obtained from surface scanners [32]. This could be explained by either the difference in resolution between CBCT (80 μm) and the model scanner (6 μm) [32] or by the software algorithms associated with the desktop scanners that yield a smooth surface after any scanning process. Finally, one of CBCT’s disadvantages affecting the image quality is the occasional noise, including the scattering radiation [32].

Our study showed a trend toward lower accuracy between IOS and desktop-scanned silicone impressions. These findings could be explained by the acquisition or stitching method found in many scanner systems [28, 33]. Scanners acquire images stitched by its software through functionality called the best-fit algorithm. To obtain acceptable stitching, the scanned object has to have adequate geometry. If the scanned area has a simple geometry, the stitching of the images could lead to deviation [28]. Typically, posterior teeth have occlusal surfaces with complex geometries and anatomical landmarks. As a result, it is easier to align these teeth-bearing areas than edentate areas or the incisal surface of the anterior teeth. Moreover, each time an additional area gets scanned and stitched with a best-fit algorithm, another source of error is added [33]. Because of this, scanning longer spans or full arch scans would increase errors, especially at the most distal ends of the scans.

## Conclusion

From our results, the following conclusions can be drawn regarding the precision of the digital models obtained from two alternative workflows to the IOS:

1. Dental and intra-arch digital measurements obtained from IOS, dental impression scanning, and dental impression CBCT scanning showed high accuracy.
2. Despite this, the linear measurements and the deviation analysis showed a trend toward less precision between the IOS and dental impressions scanning.
3. Digital models obtained from these two alternative workflows have clinically acceptable accuracy and precision of tooth size and intra-arch measurements, providing the use of high-quality devices.

## Supplementary Information

Below is the link to the electronic supplementary material.

Supplementary file1 (DOCX 22 KB)

## Data Availability

Data are available from the corresponding author upon request after approval by all the authors.
