# Supplementary material for: The precision of two alternative indirect workflows for digital model production: an illusion or a possibility?
Source: Clin Oral Investig. 2023 Apr 13;27(7):3787–97. doi: 10.1007/s00784-023-04996-2 (PMC10329612; doi:10.1007/s00784-023-04996-2)
Supplement: Supplementary file 1 — Supplementary file1 (DOCX 22 KB) [file 784_2023_4996_MOESM1_ESM.docx]

**Supplemental Table 1**

**ICC used to determine the inter-observer reliability of the repeated measurement errors**

|  | **Intraclass Correlation** | **95% Confidence Interval** | |  | |
| --- | --- | --- | --- | --- | --- |
|  |  | **Lower Bound** | **Upper Bound** | **Value** | **Sig** |
| **Anteroposterior** |  | | | | |
| Single Measures | .985 | 0.866 | 0.998 | 134.277 | 0.000 |
| Average Measures | .993 | 0.928 | 0.999 | 134.277 | 0.000 |
| **Inter-canine** |  | | | | |
| Single Measures | .955 | 0.636 | 0.995 | 43.228 | 0.002 |
| Average Measures | .977 | 0.778 | 0.998 | 43.228 | 0.002 |
| **Inter-molar** |  | | | | |
| Single Measures | .751 | -0.154 | 0.971 | 7.042 | 0.043 |
| Average Measures | .858 | -0.364 | 0.985 | 7.042 | 0.043 |
| **Vertical** |  | | | | |
| Single Measures | .948 | 0.590 | 0.994 | 37.273 | 0.002 |
| Average Measures | .973 | 0.742 | 0.997 | 37.273 | 0.002 |
| **R1** |  | | | | |
| Single Measures | .987 | 0.878 | 0.999 | 148.166 | 0.000 |
| Average Measures | .993 | 0.935 | 0.999 | 148.166 | 0.000 |
| **R2** |  | | | | |
| Single Measures | .969 | 0.735 | 0.997 | 62.920 | 0.001 |
| Average Measures | .984c | 0.847 | 0.998 | 62.920 | 0.001 |
| **R3** |  | | | | |
| Single Measures | .839 | 0.088 | 0.982 | 11.452 | 0.018 |
| Average Measures | .913 | 0.161 | 0.991 | 11.452 | 0.018 |
| **R4** |  | | | | |
| Single Measures | .908 | 0.365 | 0.990 | 20.668 | 0.006 |
| Average Measures | .952 | 0.535 | 0.995 | 20.668 | 0.006 |
| **R5** |  | | | | |
| Single Measures | .821 | 0.030 | 0.980 | 10.198 | 0.023 |
| Average Measures | .902 | 0.058 | 0.990 | 10.198 | 0.023 |
| **R6** |  | | | | |
| Single Measures | .939 | 0.535 | 0.993 | 31.710 | 0.003 |
| Average Measures | .968 | 0.697 | 0.997 | 31.710 | 0.003 |
| **Lt1** |  | | | | |
| Single Measures | .995 | 0.953 | 0.999 | 395.949 | 0.000 |
| Average Measures | .997 | 0.976 | 1.000 | 395.949 | 0.000 |
| **Lt2** |  | | | | |
| Single Measures | .773 | -0.102 | 0.974 | 7.820 | 0.036 |
| Average Measures | .872 | -0.228 | 0.987 | 7.820 | 0.036 |
| **Lt3** |  | | | | |
| Single Measures | .805 | -0.018 | 0.978 | 9.258 | 0.027 |
| Average Measures | .892 | -0.037 | 0.989 | 9.258 | 0.027 |
| **Lt4** |  | | | | |
| Single Measures | .790 | -0.059 | 0.976 | 8.529 | 0.031 |
| Average Measures | .883 | -0.126 | 0.988 | 8.529 | 0.031 |
| **Lt5** |  | | | | |
| Single Measures | .600 | -0.412 | 0.949 | 4.000 | 0.104 |
| Average Measures | .750 | -1.401 | 0.974 | 4.000 | 0.104 |
| **Lt6** |  | | | | |
| Single Measures | .868 | 0.190 | 0.985 | 14.098 | 0.013 |
| Average Measures | .929 | 0.319 | 0.993 | 14.098 | 0.013 |

**Supplemental Table 2**

**Mean difference between each two workflows in relation to dental measurements (n = 18)**

|  | **IOS vs. Scan** | **IOS vs. CBCT** | **Scan vs. CBCT** |
| --- | --- | --- | --- |
| **Maxillary** |  |  |  |
| R6 | 0.160 ± 0.062 | 0.082 ± 0.068 | -0.077 ± 0.066 |
| R5 | 0.004 ± 0.065 | 0.206 ± 0.086 | 0.202 ± 0.073 |
| R4 | 0.170 ± 0.071 | 0.086 ± 0.058 | -0.085 ± 0.082 |
| R3 | 0.029 ± 0.092 | 0.184 ± 0.160 | 0.155 ± 0.166 |
| R2 | 0.122 ± 0.071 | 0.146 ± 0.054 | 0.023 ± 0.083 |
| R1 | 0.213 ± 0.063 | 0.197 ± 0.092 | -0.016 ± 0.099 |
| L1 | 0.115 ± 0.046 | 0.125 ± 0.048 | 0.010 ± 0.064 |
| L2 | 0.219 ± 0.076 | 0.120 ± 0.062 | -0.099 ± 0.056 |
| L3 | 0.118 ± 0.094 | 0.438 ± 0.190 | 0.320 ± 0.183 |
| L4 | 0.143 ± 0.039 | 0.101 ± 0.070 | -0.042 ± 0.071 |
| L5 | 0.078 ± 0.059 | 0.169 ± 0.084 | 0.091 ± 0.080 |
| L6 | 0.279 ± 0.062 | 0.199 ± 0.092 | -0.080 ± 0.102 |
| **Mandibular** |  |  |  |
| R6 | 0.130 ± 0.087 | 0.058 ± 0.041 | -0.072 ± 0.083 |
| R5 | 0.035 ± 0.065 | 0.086 ± 0.085 | 0.051 ± 0.074 |
| R4 | 0.171 ± 0.075 | 0.018 ± 0.064 | -0.153 ± 0.093 |
| R3 | 0.121 ± 0.036 | 0.089 ± 0.094 | -0.032 ± 0.096 |
| R2 | 0.197 ± 0.085 | 0.077 ± 0.052 | -0.120 ± 0.086 |
| R1 | 0.215 ± 0.082 | 0.109 ± 0.069 | -0.106 ± 0.075 |
| L1 | 0.252 ± 0.047 | 0.200 ± 0.050 | -0.052 ± 0.075 |
| L2 | 0.227 ± 0.086 | 0.049 ± 0.059 | -0.179 ± 0.083 |
| L3 | 0.133 ± 0.076 | 0.014 ± 0.076 | -0.119 ± 0.073 |
| L4 | 0.065 ± 0.074 | 0.050 ± 0.071 | -0.015 ± 0.053 |
| L5 | 0.100 ± 0.055 | 0.121 ± 0.050 | 0.021 ± 0.053 |
| L6 | 0.022 ± 0.059 | 0.026 ± 0.067 | 0.004 ± 0.075 |

**Data was expressed using Mean ± SEM.**  SEM: **Standard error**

**Supplemental Table 3**

**Mean difference between each two workflows in relation to intra-arch measurements (n = 18)**

|  | **IOS vs. Scan** | **IOS vs. CBCT** | **Scan vs. CBCT** |
| --- | --- | --- | --- |
| **Maxillary** |  |  |  |
| Right AP dimension | 0.217 ± 0.153 | -0.026 ± 0.135 | -0.243 ± 0.081 |
| Inter-canine | 0.272 ± 0.171 | 0.044 ± 0.136 | -0.228 ± 0.085 |
| Inter-molar | 0.382 ± 0.099 | 0.077 ± 0.110 | -0.305 ± 0.099 |
| Right vertical dimension | 0.389 ± 0.142 | 0.578 ± 0.171 | 0.188 ± 0.095 |
| **Mandibular** |  |  |  |
| Right AP dimension | 0.275 ± 0.138 | 0.203 ± 0.150 | -0.072 ± 0.106 |
| Inter-canine | 0.484 ± 0.216 | 0.239 ± 0.189 | -0.245 ± 0.112 |
| Inter-molar | 0.421 ± 0.255 | 0.112 ± 0.286 | -0.308 ± 0.098 |
| Right vertical dimension | 0.244 ± 0.112 | 0.557 ± 0.153 | 0.312 ± 0.123 |

**Data was expressed using Mean ± SEM.**  SEM: **Standard error**

**Supplemental Table 4**

**Mean deviation for the studied groups**

|  | **Mean (mm)** | **SD** |
| --- | --- | --- |
| **CBCT imp Vs Scan imp** | -0.09 | 0.03 |
| **IOS Vs CBCT imp** | -0.08 | 0.06 |
| **IOS Vs Scan imp** | -0.1 | 0.08 |

**Supplemental Table 5**

**The mean and standard deviation of the inter-observer linear and dental measurements**

|  | **Minimum** | **Maximum** | **Mean** | **Std. Deviation** |
| --- | --- | --- | --- | --- |
| **Anteroposterior** |  | | | |
| Examiner 1 | 20.37 | 23.76 | 21.9040 | 1.57303 |
| Examiner 2 | 20.75 | 23.83 | 22.0372 | 1.60527 |
| **Inter-canine** |  | | | |
| Examiner 1 | 31.53 | 33.82 | 32.6100 | 0.97008 |
| Examiner 2 | 31.60 | 33.78 | 32.5946 | 0.91091 |
| **Inter-molar** |  | | | |
| Examiner 1 | 49.68 | 51.07 | 50.2760 | 0.61248 |
| Examiner 2 | 50.23 | 51.10 | 50.6626 | 0.41521 |
| **Vertical** |  | | | |
| Examiner 1 | 8.33 | 9.12 | 8.7900 | 0.30455 |
| Examiner 2 | 8.61 | 9.58 | 9.0852 | 0.35681 |
| **R1** |  | | | |
| Examiner 1 | 4.80 | 7.35 | 6.7438 | 1.09424 |
| Examiner 2 | 4.80 | 7.15 | 6.6368 | 1.02796 |
| **R2** |  | | | |
| Examiner 1 | 5.41 | 5.63 | 5.5188 | 0.09510 |
| Examiner 2 | 5.34 | 5.63 | 5.4960 | 0.11760 |
| **R3** |  | | | |
| Examiner 1 | 6.43 | 7.23 | 7.0040 | 0.32647 |
| Examiner 2 | 6.43 | 6.89 | 6.7520 | 0.18365 |
| **R4** |  | | | |
| Examiner 1 | 5.02 | 5.57 | 5.2396 | 0.21239 |
| Examiner 2 | 5.12 | 5.55 | 5.2962 | 0.22055 |
| **R5** |  | | | |
| Examiner 1 | 5.57 | 6.28 | 5.9952 | 0.33488 |
| Examiner 2 | 5.61 | 6.90 | 6.2578 | 0.59272 |
| **R6** |  | | | |
| Examiner 1 | 8.63 | 9.92 | 9.0316 | 0.51547 |
| Examiner 2 | 8.75 | 9.89 | 9.1192 | 0.47023 |
| **Lt1** |  | | | |
| Examiner 1 | 4.90 | 7.32 | 6.7456 | 1.03371 |
| Examiner 2 | 4.90 | 7.13 | 6.6296 | 0.96785 |
| **Lt2** |  | | | |
| Examiner 1 | 5.26 | 6.02 | 5.5744 | 0.28195 |
| Examiner 2 | 5.15 | 5.59 | 5.3288 | 0.16910 |
| **Lt3** |  | | | |
| Examiner 1 | 6.42 | 7.37 | 6.8976 | 0.37216 |
| Examiner 2 | 6.42 | 7.05 | 6.7464 | 0.26066 |
| **Lt4** |  | | | |
| Examiner 1 | 5.51 | 5.78 | 5.6254 | 0.10869 |
| Examiner 2 | 5.56 | 5.79 | 5.6622 | 0.08446 |
| **Lt5** |  | | | |
| Examiner 1 | 5.69 | 5.93 | 5.8504 | 0.09533 |
| Examiner 2 | 5.71 | 5.87 | 5.7950 | 0.07824 |
| **Lt6** |  | | | |
| Examiner 1 | 8.89 | 9.83 | 9.1362 | 0.39644 |
| Examiner 2 | 8.61 | 9.81 | 9.1004 | 0.45870 |
